# Recent advances in therapeutic CRISPR-Cas9 genome editing: mechanisms and applications

**DOI:** 10.1186/s43556-023-00115-5

**Published:** 2023-04-07

**Authors:** Lifang Zhou, Shaohua Yao

**Affiliations:** Laboratory of Biotherapy, National Key Laboratory of Biotherapy, Cancer Center, West China Hospital, Sichuan University, Renmin Nanlu 17, Chengdu, 610041 Sichuan China

**Keywords:** CRISPR/Cas9, Base editing, Prime editing, Gene therapy, Gene Delivery

## Abstract

Recently, clustered regularly interspaced palindromic repeats (CRISPR)-Cas9 derived editing tools had significantly improved our ability to make desired changes in the genome. Wild-type Cas9 protein recognizes the target genomic loci and induced local double strand breaks (DSBs) in the guidance of small RNA molecule. In mammalian cells, the DSBs are mainly repaired by endogenous non-homologous end joining (NHEJ) pathway, which is error prone and results in the formation of indels. The indels can be harnessed to interrupt gene coding sequences or regulation elements. The DSBs can also be fixed by homology directed repair (HDR) pathway to introduce desired changes, such as base substitution and fragment insertion, when proper donor templates are provided, albeit in a less efficient manner. Besides making DSBs, Cas9 protein can be mutated to serve as a DNA binding platform to recruit functional modulators to the target loci, performing local transcriptional regulation, epigenetic remolding, base editing or prime editing. These Cas9 derived editing tools, especially base editors and prime editors, can introduce precise changes into the target loci at a single-base resolution and in an efficient and irreversible manner. Such features make these editing tools very promising for therapeutic applications. This review focuses on the evolution and mechanisms of CRISPR-Cas9 derived editing tools and their applications in the field of gene therapy.

## Introduction

Genome editing has become a powerful tool for both basic biomedical research and translational medicine. The development of reprogrammable nucleases, especially clustered regularly interspaced short palindromic repeats (CRISPR)-Cas9 system, has revolutionized the field of genome editing [1, 2]. Since the discovery demonstrated that the wild-type (WT) Cas9 system produced double strand breaks at target region in vitro in the guidance of a pair of small RNA molecules, crRNA and tracrRNA, which were later engineered to a single guide RNA (sgRNA) [1, 2], the system soon found its applications in multiple organisms and in a wide range of scenarios [3–9]. In eukaryotic cells, Cas9 can be used to induce double strands breaks (DSBs) in the genome, which are frequently repaired by non-homologous end joining (NHEJ) pathway since NHEJ is more active than other DNA repair systems in repairing DSBs, leading to the formation of insertion or/and deletion (indel) mutations [5, 10].

Besides producing DSB, Cas9 could also be engineered to renounce the ability of DNA cleavage but reserve the ability of DNA binding, thereby serving as a platform for recruiting other DNA regulators or modifiers to induce localized transcriptional, epigenetic or genetic manipulations. To the best of our knowledge, up to date the platform has been utilized to develop transcriptional regulators [11–17], histone methylation [18–20] or acetylation modifiers [17, 21], DNA methylation modifiers [19], cytosine or adenine base editors [22–26] and primer editors [25, 27–29]. Among these tools, base editors and primer editors have the potential for clinical translation because both of them can produce permanent and desired changes in genomic sequences [22, 26, 27, 30, 31]. By recruiting deaminases, base editors produce targeted deamination on cytosines (Cs) or adenines (As) dependending on the types of deaminases, which in turn are converted into thymines (Ts) or guanines (Gs) respectively by endogenous DNA repair or replication mechanisms [22, 23, 32–35]. Prime editors consist of a Cas9 nickase and reverse transcriptase (RT) fusion protein and a prime editing guide RNA (pegRNA). When binding to target DNAs, the Cas9 nickase introduced a nick in the non-target strand (NTS), which is then bound by the primer binding sequences (PBS) of pegRNA [27]. RT recognized the DNA/RNA duplex and extended the ssDNA under the guidance of RT template of the pegRNA [27]. Finally, the information of the extended ssDNA is written into the genome through endogenous DNA repair mechanisms to accomplish the editing [27, 36–38].

These editing tools, together with Cas9 nuclease, enable virtually any desired changes in the genome, therefore have significant potential for clinical applications for disease treatment. Cas9 derived tools have achieved significant success in animal models of human genetic disease as well as cancer, either via inactivating the expression or function of specific genes [39–43], correcting disease relevant mutations to restore gene function [44–48], or reactivating the expression of functional redundant genes to compensate for mutant genes [49–52]. With the development of in vivo delivery system, it is now practicable to efficiently deliver Cas9 derived tools into multiple target organs, including liver, eye, ear and muscles etc. Up to date, more than 34 clinical trials using Cas9 tools have been recruited, among which several trials have reported therapeutic benefit for patients, highlighting the potential of Cas9 tools in clinical translation. Here, we will introduce recent advances in the evolution and mechanisms of CRISPR-Cas9 derived editing tools, focusing on the stepwise actions of these tools and the responses of endogenous DNA repair pathways. In addition, we will also introduce their applications in the field of gene therapy, with special emphases on the design of editing strategy and the in vivo delivery of these tools.

## Cas9 and its derivates

### CRISPR system

Reprogrammable nucleases that target DNA molecules are the fundamental players in genome editing, which introduce targeted cleavage of specific DNA sequences. To date, four classes of DNA nucleases that possess genome editing potential have been discovered, which were meganucleases, zinc finger nucleases (ZFNs) [53, 54], transcription activator–like effector nucleases (TALENs) [55–57] and CRISPR-associated nucleases [58]. Distinct from the former three nucleases that recognize DNA through sequence-specific protein-DNA interaction, Cas9s utilized a bi-interaction modes to recognize their target DNA, i.e., protein-DNA and RNA–DNA interactions [1, 2].

CRISPR system was first discovered in bacteria genome, where regular sequences consisting of repeat sequences and spacer sequences were found [59]. Later, it was demonstrated to function as a bacterial defense system against invaded foreign DNAs [60]. Upon the invasion of foreign DNAs, such as plasmids and phages, the CRISPR system is activated to cut a small piece of invaded DNA and insert it into the CRISPR locus of bacterial genome [60] The inserted foreign DNAs are then transcribed, together with the sequences from the host genomes, to form fusion RNAs that was named CRISPR repeat RNA (crRNA), which guides CRISPR proteins to cleave the invaded DNAs [2, 61–65].

#### Cas9 nuclease

In 2012, Jenniffer Doundna and Emmanuelle Charpentier labs provided the first in vitro data demonstrating that Cas9 system derived from Streptococcus pyogenes can be re-engineered to recognize new target DNA [1]. They showed that changing the protospacer sequences of crRNA can guide the Streptococcus pyogenes Cas9 (SpCas9) to recognize target DNA that contains the spacer sequences in front of a motif called protospacer adjacent motif (PAM). By recognizing the target DNA, SpCas9 cleaved both DNA strands 3 nucleotides upstream the PAM, leading to double strand break (DSB). Several months later, Virginijus Siksnys lab reported another Cas9 system, from Streptococcus thermophilus, also capable of introducing in vitro DSB in a reprogrammable manner (Cas9-crRNA ribonucleoprotein complex mediates specific DNA cleavage for adaptive immunity in bacteria) [2]. Soon after these pioneer works, Feng Zhang and George Church labs independently demonstrated that this system can be functionally introduced into mammalian cells to target a large variety of genomic loci [4, 5]. In mammalian cells, DSBs produced by Cas9, or other nucleases are mainly repaired by an error-prone DNA repair mechanism, named non-homologous end joining (NHEJ) pathway, resulting in the formation of insertion and deletion (indel) mutations [4, 5, 66].

**Fig. 1:**
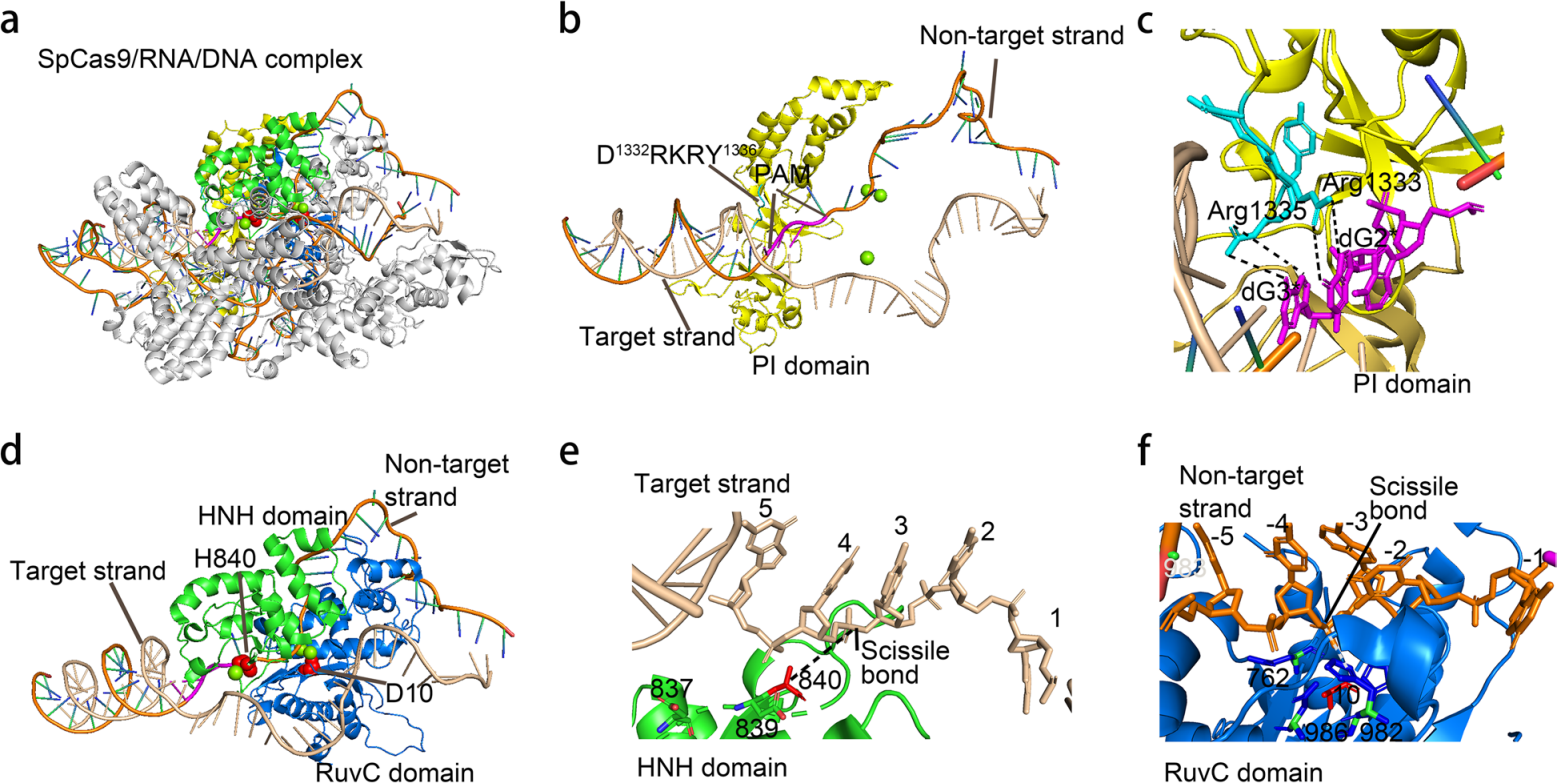
Structure of spCas9/sgRNA/DNA complex. **a** Overall structure of spCas9/sgRNA/DNA complex, with PI, HNH and RuvC domains shown in yellow, green and blue respectively. **b** and **c** Detailed structure showing the interaction between PI domain and PAM (**b**), with key amino acids highlighted in pink (**c**). **d** Detailed structure showing the HNH domain, RuvC domain and target DNA, with key amino acids (D10 and H840) shown in sphere. Mutation of Asparticacid 10 (D10) or Histidine 10 (H840) to Alanine (A) silences the RuvC or the HNH domain respectively, resulting in TS nickase or NTS nickase respectively. Mutation of both amino acids silences both domains, resulting in catalytic dead Cas9. **e** Detailed structure showing the key amino acids interaction with target strand in HNH domain. **f** Detailed structure showing the key amino acids interaction with target strand in RuvC domain

Following these works, Cas9s from different microorganisms were investigated. These efforts generated a growing list of Cas9 systems that were capable of editing eukaryotic genomes, including SaCas9 [39, 67–69], CjCas9 [70–73], NmCas9 [74–77], FnCas9 [78–81] and SauriCas9 [82, 83] etc. Importantly, these Cas9s recognize different PAMs, which increases the targeting scope of Cas9 based editing strategies (Table 1). Structurally, all Cas9 proteins studied so far exhibit bilobed architecture that consist of one recognition (REC) and one nuclease (NUC) lobes [84]. Three major functional domains were identified, including two nuclease domains, RuvC and HNH, and one PAM-interacting domain (PI domain) (Fig. 1). The PI domain, usually locating in the C-terminus of Cas9 proteins, recognizes the PAM sequences. Upon recognizing PAM, Cas9 sharply bends and undertwists DNA to flip DNA nucleotides out of the duplex and toward the sgRNA. Once the spacer region of the target DNA matched the sgRNA guide sequences, the sgRNA invades into the DNA duplex to partially detached the nontarget strand (NTS), thereby forming an “R-loop” structure [85–89]. The binding of Cas9 protein to the target DNA induced conformational changes in Cas9 structure, which triggers its cleavage activity [84, 87]. The HNH and RuvC nuclease domains of Cas9 protein are responsible for cleavage of the complementary and noncomplementary strands of the target DNA, respectively [84]. Noteworthy, the cleavage of DNA strand is independent to each other [84]. Therefore, Cas9 proteins can be mutated into nickases that nicked either TS or NTS or dead proteins without any cleavage activity [90].

**Table 1:** Cas9 and their respective PAM sequences

Cas9 variants	Organisms	PAM	Reference
SpCas9	*Streptococcus pyogenes*	NGG	[1, 4, 5]
SpCas9-EQR	*Streptococcus pyogenes*	NGAG	[91]
SpCas9-VQR	*Streptococcus pyogenes*	NGA	[91]
SpCas9-VRER	*Streptococcus pyogenes*	NGCG	[91]
xCas9	*Streptococcus pyogenes*	NG; NNG	[92]
Cas9-NG	*Streptococcus pyogenes*	NG	[93]
SpG	*Streptococcus pyogenes*	NG	[94]
SpRY	*Streptococcus pyogenes*	NR; NY	[94]
ScCas9	*Streptococcus canis*	NNG	[95]
FnCas9	*Francisella novicida*	NGG	[78, 79]
S. thermophilus CRISPR1	*Streptococcus thermophilus*	NNRRRA	[39, 96]
S. thermophilus CRISPR3	*Streptococcus thermophilus*	NGGNG	[2]
FrCas9	*Faecalibaculum rodentium*	NNTA	[97]
SaCas9	*Staphylococcus aureus*	NNGRRT	[39]
SaCas9-KKH	*Staphylococcus aureus*	NNNRRT	[98]
SauriCas9	*Staphylococcus Auricularis*	NNGG	[82]
SlugCas9	*Staphylococcus lugdunensis*	NNGG	[83]
SchCas9	*Staphylococcus chromogenes*	NNGR	[99]
CjCas9	*Campylobacter jejuni*	NNNNRYAC	[70, 100]
Nm1Cas9	*Neisseria meningitidis*	NNNNGATT	[74, 101]
Nm2Cas9	Neisseria meningitidis	NNNNCC	[102]
GeoCas9	Geobacillus stearothermophilus	NNNNCRAA	[103]
Glc300Cas9	Geobacillus LC300	NNNNGMAA	[103]
CcCas9	Clostridium cellulolyticum	NNNNGNA	[104]
CdCas9	Corynebacterium diphtheriae	NNRHHHY	[105]

#### Cas12 nuclease

In addition to Cas9, other CRISPR systems were also found to be capable of editing eukaryotic genomes. Up to date, three major types of CRISPR systems were identified, type I-III. They were classified according to their components and action mechanisms [106]. Among these systems, Cas12a (also named Cpf1, hereafter referred as to Cpf1) proteins from type II CRISPR were also frequently used in genome editing because of its simplicity of action and easiness to be reprogrammed, since this system consists of a single nuclease protein and a single RNA component [107–110]. Like Cas9, Cpf1 recognizes its target DNA also through the two types of interactions, i.e., RNA/DNA and protein/DNA [111]. However, compared with Cas9, Cpf1 protein can only be mutated to form NTS nickase or dead Cpf1 but not TS nickase, because the activation of TS cleavage is triggered by the cleavage of NTS [111]. Therefore, Cpf1 protein is less versatile than Cas9.

#### Engineered Cas9 variants with expanded targeting scope

As PAM recognition is a key limitation for the targeting scope of Cas9 proteins, many efforts have been made to engineer them to relax the limitation. As mentioned above, structural investigations have revealed that PI domain is responsible for sequence-specific binding of PAM [84, 112] (Fig. 1a, b, c). Under the guidance of structural information, several Cas9 variants had been designed harboring mutations within PI domain to change the PAM preference. Most efforts have been put on SpCas9, leading to the discovery of a panel of SpCas9 PI variant, including Cas9-VQR [91], Cas9-EQR [91], and Cas9-VRER [91], Cas9-NG [93], Non-G SpCas9s [113], SpG and SpRY [94]. Among these variants, Cas9-VQR, Cas9-EQR, and Cas9-VRER changed the PAM specificity from NGG to NGA, NGAG, or NGCG, respectively [91]. And Non-G SpCas9s recognize NRNH PAMs (where R is A or G and H is A, C or T) [113]. Other variants, including Cas9-NG, SpG and SpRY, relaxed the PAM restriction from NGG to NGN (Cas9-NG and SpG) and NRN or NYN (SpRY, NRN > NYN) respectively [93, 94]. Interestingly, another variant, xCas9, also significantly relaxed the PAM restriction by recognizing NGN, NNG, GAA, GAT, and CAA PAMs. However, the majority of mutated amino acids of xCas9 did not locate in the PI domain (six out of seven), suggesting that other structures beyond PI domain also contribute to PAM recognition. Interestingly, coupling non-PI domain mutations of xCas9 to PI domains of Cas9-NG or Non-G Cas9 variants also relaxed the PAM restriction of the latter. Collectively, these variants significantly expanded the targeting scope of Cas9 systems [92].

#### Genome editing produced by wild-type Cas9

**Fig. 2:**
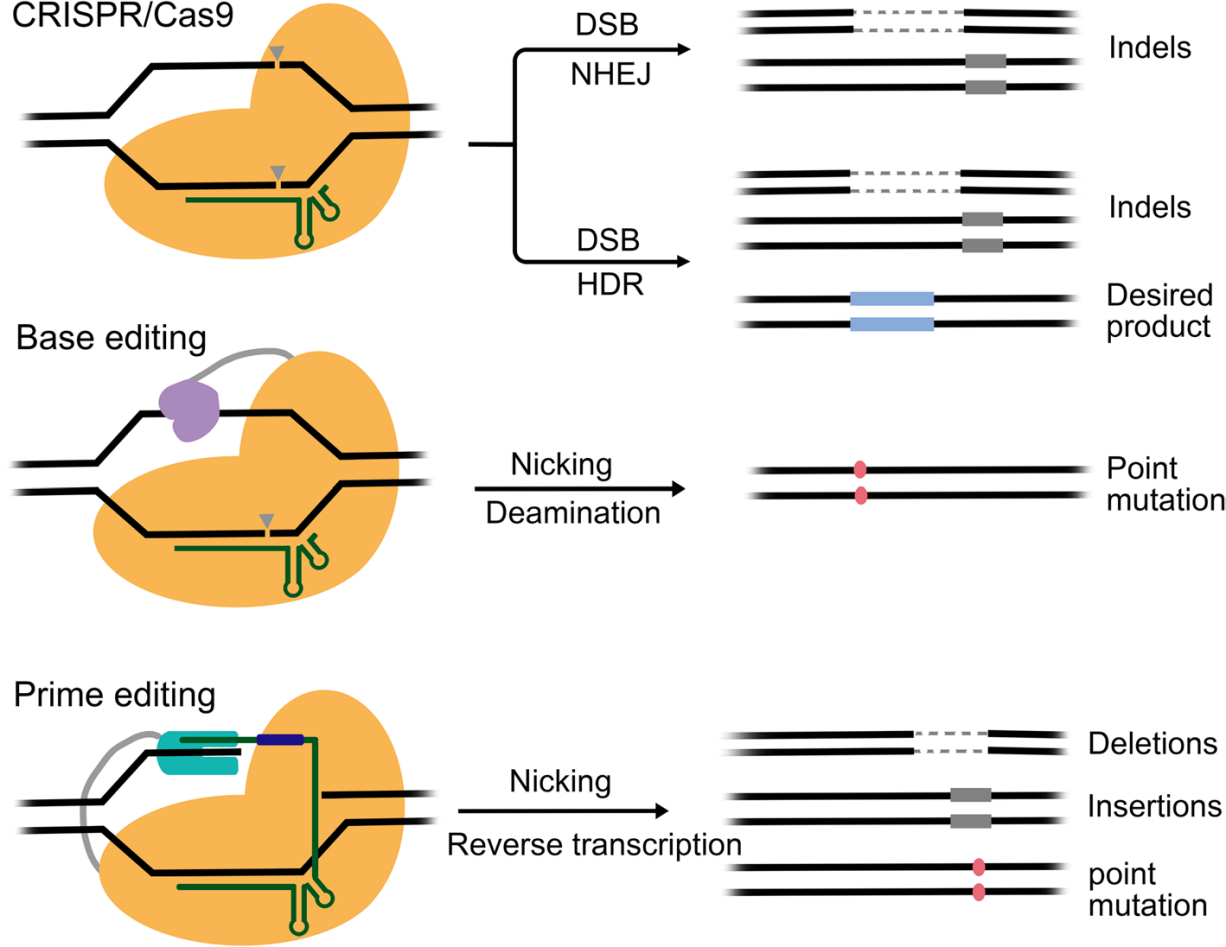
Editing processes outcomes of Cas9 nuclease, base editor and prime editor

As mentioned above, in eukaryotic cells, DSBs tend to be repaired by error-prone NHEJ pathway, leading to the formation of uncontrolled indels [10] (Fig. 2). Such feature can be utilized to disrupt functional elements of the genome to manipulate gene functions. For example, introducing indels into gene coding regions can change the codon or even shift the reading frame, thereby disrupting gene function [39, 43, 114, 115]. Indels can also be introduced to the boundary regions between exons and introns to interrupt RNA splicing or to the enhancers or suppressors to change the expression pattern of specific genes [6, 116, 117]. In addition, when supplied with donor DNAs harboring sequences homologous to each end of DSBs, they can also be repaired by homology dependent mechanisms, such as homology directed repair (HDR) pathway [44, 118–121] or MMEJ [122–126] (Fig. 2). However, the efficiency of HDR pathway is much lower than that of NHEJ [10]. Because NHEJ is very active in the whole cell cycle and can be observed in various cell types, including cells in division and cells after mitosis. In contrast, HDR mainly plays a role in the S/G2 stage, so it is mainly limited to actively dividing cells, limiting the treatment that requires precise genomic modification of mitotic cells [127, 128].

### Base editor

Besides functioning as nuclease, Cas9 proteins can also be rewired to function as DNA binding platforms to recruit other functional modulators. By linking with modulators such as transcription regulators and histone or DNA modifiers, Cas9s can be engineered to regulate transcription, histone and DNA modifications [11, 12, 21]. Among these derivates, base editors, in which dead or nickase Cas9 proteins were fused to cytosine (C) or adenine (A) deaminase (CBE or ABE), are the most interesting ones in that they can efficiently and precisely convert one target DNA base pair into another [22, 30] (Fig. 2).

As above mentioned, Cas9 proteins bind to their target DNAs to form an “R-Loop” structure, in which the NTS was partially detached and exposed outside the Cas9 complex [88]. The exposed NTS is a favorite target for C or A deaminase that prefers single-strand DNA [129]. Usually, the deaminases are fused to the N-terminus of Cas9 protein, thereby deaminating Cs or As that locate in a small window of NTS, called editing window [22, 30]. Typically, SpCas9 derived N-terminal base editors function in a window ranging from ~ 4 to ~ 8 bases, counting NGG PAM as 21–23. These Cs or As in the window were deaminated into urines (Us) or inosines (Is), which in turn are transformed into thymines (Ts) or guanines (Gs) by endogenous DNA repair or replication mechanisms [22, 30]. Reserving the cleavage activity of Cas9 for TS significantly increased the transformation efficiency, which is possibly due to the nick within TS induce endogenous mechanisms to fix the lesion according to the unbroken NTS [130].

#### Editing window of base editor

The width and position of the editing window varied with the types and locations of deaminases. For example, deaminases with decreased enzyme activity tend to narrow the editing window [131]. And inlaying or tethering deaminases to different locations of the Cas9 complex shift the position of the editing window [132–136]. By contrast, tethering multiple copies of deaminases to base editor enlarges the editing window [137]. By adjusting these parameters, now we have a large variety of editors with different editing windows [138–141]. In addition, reducing the length of sgRNA spacer has also been shown to narrow the editing windows in some cases [142–144]. It is also noteworthy that when equipped with both deaminases, base editors can achieve simultaneous conversion of both cytosine and adenine, which is particularly useful for saturated mutation screening [132, 145–147].

#### Off-target editing of base editor

Although base editors are efficient in on-target editing, they also produce undesired off-target editing on both DNA and RNA molecules [148–152]. Basically, the off-target effects of base editors can be classified into sequence (or Cas9) dependent [153–157] and independent [150, 152] effects. Sequence dependent off-target editing mainly occurred at the genomic DNAs, including Cas9 off-target genomic loci or the on-target locus but outside the editing window [148, 158]. Sequence independent off-target editing took place in both DNA and RNA molecules [149, 150, 152]. Since the deaminases prefer single strand nucleic acids, RNAs and occasionally occurred single strand DNAs can be their substrates [149]. These off-target effects are suspected to lead to pathologic conditions, raising the concern of safety issue. To resolve this issue, many efforts have been put to reduce the off-target base editing. Firstly, deaminases with reduced enzyme activity have been shown to significantly decrease the off-target efficiency while has little effects on the on-target efficiency [134, 148, 159, 160]. Secondly, inlaying deaminases into Cas9 protein specifically reduced the sequence independent RNA and DNA off-target editing [136, 161]. Moreover, splitting the deaminases to timely control their activity also helps reduce off-target editing, which is particularly useful in long-term delivery of base editors, such as AAV delivery [162, 163].

#### C to G base editing

It is noteworthy that base editors, especially CBEs, frequently produce impure editing outcomes, including indels and C to R conversions [30] (*R* = A or G). This is possibly because U within DNA molecules tend to be removed by endogenous uracil-N-glycosylase (UNG), since impure products were nearly absent in UNG null cells [23]. UNG cleaves the N-glycosidic bond of uracil and leaves an abasic site (also known as apurinic/apyrimidinic site, AP site) [164–170]. The resulting abasic site can be recognized by a base excision repair enzyme, AP lyase, which converts abasic site to ssDNA break [167, 171–177]. Together with the nick within the target strand that is cleaved by Cas9 nickase, they were likely recognized as DSB by DNA repair machines, leading to the formation of indels. Alternatively, the abasic site is not transformed into nick, enabling NTS to serve as template to guide the repair of the TS. The repair of the TS might involve a trans-lesion synthesis pathway that uses a specialized DNA polymerase to bypass the lesion by adding a special nucleotide opposite to the abasic site [164, 177–179]. It is likely that the repaired TS may in turn serve as a template to guide the repair of NTS to fix the abasic site. Thereby, the trans-lesion synthesis pathway introduces an R base to substitute the deaminated C. The level and the type of C to R conversion are likely dependent on the species and sequence context where the deaminated C locates [180]. In mammalian cells, U s tends to be converted to Gs while in bacteria they tends to be converted to As [181–185]. Supplementing additional copies to CBE may further inhibit the endogenous UNG activity, thereby increasing the purity of the editing products by reducing the frequencies of C to R conversion or indel formation [23, 186, 187]. Unlike CBEs, ABEs rarely create impure editing outcomes, which is possibly due to that the activity of endogenous pathways dealing with inosine lesions is rather low [188]. Although ABEs also produce ssDNA breaks in the TS and ssDNA breaks themselves induce the formation of indel, the level of indels in ABE products are generally low (in most cases, < 1%) [189–191].

### Prime editor

Base editing can efficiently install base transition mutations, i.e. the substitutions in-between purines or pyrimidines (from A to G, G to A, C to T or T to C), making it possible to correct human pathogenic diseases caused by such mutations [22, 30]. However, there were a large portion of genetic lesions caused by other types of mutation, such as base transversion, small fragment insertion and deletion etc., all of which were beyond the scope of base editing tools [192].

To fill above gaps and achieve more efficient, precise and flexible DNA changes, David Liu and co-workers proposed a new “search-and-replace” genome editing technology, called prime editing system [27]. In this system, Cas9 harboring H840A mutation was coupled with an engineered reverse transcriptase, M-MLV, to form prime editor (PE) that can achieve all 12 types point mutations, small fragment insertion and deletion (Fig. 2). Upon the guidance of prime editing guide RNA (pegRNA), PE fusion protein bind to the targeted DNA, nicked the NTS and then extend the 3’ end of NTS according to the information encoded by pegRNA [27]. Through these stepwise actions, PE incorporated new DNA sequences into the nicked NTS, which were then passed to its complementary strand, possibly through DNA repair or replication mechanisms. Thereby, PE accomplished the rewrite of the genetic information within the target site. The most beautiful part of PE system is the design of pegRNA that was composed of a sgRNA and a 3’end extended tail [27]. The 3’ tail of pegRNA contained two parts. One part was designed to complement to the 3’end of nick NTS, called primer binding site (PBS) and the other one encoding the desired edit served as reverse transcriptional template (RT-template). Once the NTS was nicked by nCas9 H840A, the 5’ portion of NTS was recognized by PBS to form a DNA-RNA duplex, which served as a good substrate for reverse transcriptase. The 5’ portion of NTS acting as a primer was extended by reverse transcriptase according the guidance of RT-template (Fig. 2).

#### Editing efficiency improvement of prime editor

Although PE system can install almost all types of DNA changes, it is overall less efficient than BE or Cas9 nuclease, which may in part due to that PE system required stepwise and concerted actions of multiple modulators [27]. Optimizing the parameters of nearly all steps is necessary to maximize the efficiency of PE. As mentioned above, the starting process of PE is to nick the NTS of the target site and bind its end with PBS. Hence, parameters enhancing this process may include the activity of Cas9 nickase and the thermodynamics of the PBS. As a matter of fact, David Liu’s lab has observed that mutations enhancing the activity of Cas9 nuclease also enhance the efficiency of PE. Caixia Gao’s lab observed that the length and sequence of PBS has a significant effect on the efficiency of PE, which is possibly related to the annealing temperature of PBS [193]. Moreover, the 3’ end of pegRNA, mainly the PBS, was thought to tend to be degraded in cells, resulting in the failure of pegRNA to prime the editing [194]. Interestingly, another line of evidence showed that the presence of PBS disrupted the structure of pegRNA by binding to its spacer sequence, resulting in the loss-of-function of pegRNA [195]. To sum up, both studies found that coupling an RNA motif harboring stem-loop structure improved the efficiency of PE. This finding was soon confirmed by other studies showing that coupling complicated structure to the 3’ end of pegRNA helped increasing the efficiency of PE [196, 197]. Enhancing the activity of RT provide another way to improve PE [198, 199]. Wen Xue’s lab showed that the removal of RNase H domain from RT improved PE [199]. Besides strengthening the elements of PE system, optimizing the design of PE with paired pegRNAs also improve the efficiency of PE, especially in cases of multiplex base conversion, large fragment deletion and insertion [28, 200–203]. Interestingly, paired pegRNA strategy also significantly decreased the frequency of undesired indels [28, 203]. In addition, modulating the endogenous mechanisms that are involved in each process of PE may affect its efficiency. The first evidence of such mechanism came from David Liu’s and Joanna Loizou’s labs [37, 38]. They independently demonstrated that the inhibition of mismatch repair pathway enhanced the efficiency and accuracy of PE. Importantly, David Liu’s lab discovered a set of enhanced prime editors by coupling dominant negative MLH1 to PE, which is a key player in the recognition of small insertion/deletion loops during the process of MMR [37]. In the evolved version of prime editors, called PE4 or PE5, dominant negative MLH1 was co-expressed with but not physically attached to PE2, resulting in the global inhibition of MLH1 activity and improved PE efficiency and decreased indel frequency [37]. Hopefully, in the near future we will see further improvements in the efficiency and precise of PE system.

### CRISPR-related transposon and recombination systems

#### CRISPR-related transposon system

Targeted insertion of large DNA fragments into desired genomic loci holds great potential for the treatment of genetic diseases. Although such kind of insertions can be achieved by wild-type Cas9 mediated HDR or micro homologous recombination (MMEJ), the efficiencies are generally low and are limited by the cell type to be edited [204, 205]. Prime editor is also capable of inserting large DNA fragments into desired loci, but its efficiency is even lower [28, 206, 207]. Therefore, efficient targeted insertion of large DNA fragments into genome remains challenging.

**Fig. 3:**
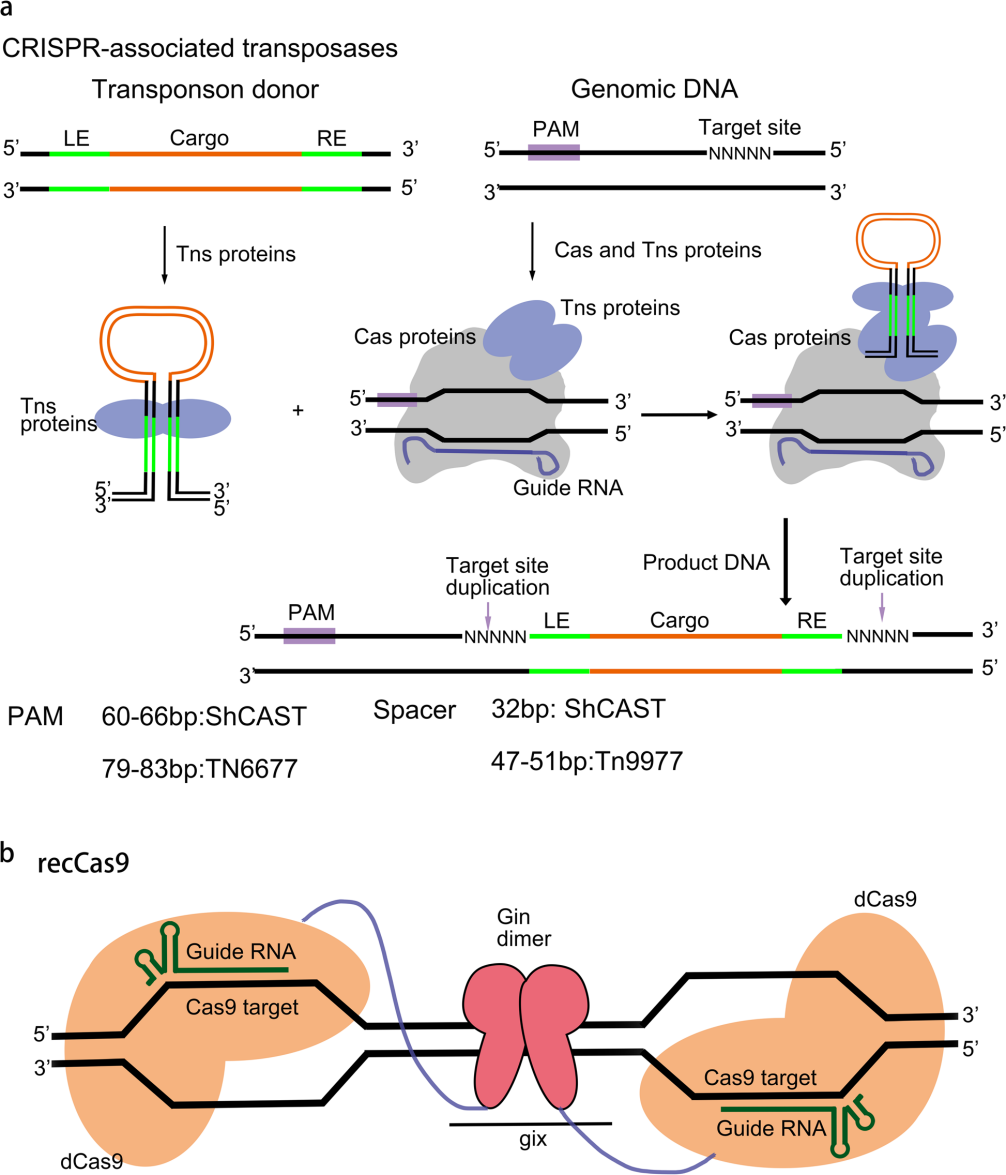
CRISPR-associated transposon and recombination systems. **a** Cas transposases include both Cas proteins and transposase-associated components. Cargo DNA is identified by its left end (LE) and right end (RE) sequences and bound by transposase proteins (Tns). Guide RNA binding to Cas nuclease brings transposase to the specific site, and transposase integrates the DNA cargo into the target site. The target site is duplicated and flanks the integrated LE–cargo–RE sequence. Each Cas-transposase complex has a specific requirement for the spacer length of guide RNA and a unique position preference of integration site. **b** reassembly of guide RNA-programmed recCas9 at the target sites

Transposition is a special type of genetic recombination, which transfers specific genetic factors from one location to another by using the interaction of transposon elements and transposase enzymes [208]. In 2017, Peters et al. found a novel kind of CRISPR Cas system encoded by a Tn7 like transposon, which consists of several key proteins homologous to the core elements of Tn7 transposons, including two isomerase (TnsA and TnsB) and one regulatory protein (TnsC), and a set of CRSIPR system [209]. This system can process and bind crRNA but cannot cut target DNA. Instead, it can achieve gene transposition at specific sites [209]. In 2019, Klompe et al. used a three-plasmid system to express CRISPR transposase derived from Vibrio cholerae in Escherichia coli for the directional integration of large pieces of DNA [210]. In 2020, to simplify the process and improve the integration efficiency, the same researchers effectively combined key elements to construct INTEGRATE single particle system pSPIN, which uses a single promoter to simultaneously drive guide RNA and multi cis–trans mRNA to express key proteins and guide RNA [211]. It is worth noting that pSpin-mediated DNA transposition has no directionality, and the rotator can be inserted in both positive and negative directions. At the same time in 2019, Strecker, et al. have developed a similar CRISPR transposase system. Researchers have developed and used the CRISPR-related transposase (CAST) of the cyanobacteria Scytonema hofmanni, which is composed of Tn7-like transposase subunit and V-K CRISPR (Cas12k). CAST includes two variants: Scytonema hofmanni (ShCAST) and Anabaena cylindrica (AcCAST). The insertion of ShCAST mainly occurs between 60 and 66 bp 3 ' of PAM, while the insertion of AcCAST cargo into 49–56 bp 3 ′ of PAM. The gene insertion mediated by ShCAST is directional [212]. In the study, after the author replaced Cas12k with Cas9, no displacement was observed, indicating that Cas12k may have a specific role with other CAST components to facilitate the occurrence of transposition [212]. Since then, Chen et al. have developed a new synthesis system, Cas Transposon (CasTn), which combines the DNA integration capability of Himar1 transposase and the programmable genome targeting capability of dCas9 to achieve targeted gene transposition. The author has proved this in cell-free in vitro reactions and E. coli plasmid detection. With the further improvement of the system, CasTn may play a role in various organisms, because the Himar1-dCas9 protein does not need host factors to play a role [213]. Noteworthy, although all the above transposon systems have good gene integration ability in E. coli, there is lacking evidence demonstrating that these systems can also function efficiently in eukaryotic cells (Fig. 3a).

#### CRISPR-related recombination systems

Site-specific recombinase directly catalyzes the cleavage, chain exchange, and coordination of two double-stranded DNA sequences, to cause gene recombination phenomena such as insertion, deletion, or inversion of the target sequence [214–217]. Unlike DNA nuclease, the direct catalysis of recombinase usually does not trigger the error-prone DNA repair process, leading to the formation of indel. It does not rely on the endogenous cell DNA repair mechanism, and the recombinant product is relatively simple [214, 215]. Therefore, recombinant enzyme-mediated genome modification can produce more accurate and predictable genome changes than ribozyme-based genome editing and may be more efficient in non-dividing cells [216, 217]. Tyrosine and serine recombinases such as Cre, Flp, and Φ C31 integrase has been widely used to catalyze the recombination of exogenous DNA into model organisms [217, 218]. However, the use of these enzymes is limited by their inherent, nonprogrammable DNA sequence specificity. Chaikind et al. fused dCas9 into the catalytic domain of Gin recombinase β and the resulting recCas9 system. This “recCas9” system shows moderate efficiency on the plasmid matrix and can mediate a large number of genome deletions in mammalian cells with low efficiency [219] (Fig. 3b).

## Therapeutic applications of Cas9 and its derivates

**Fig. 4:**
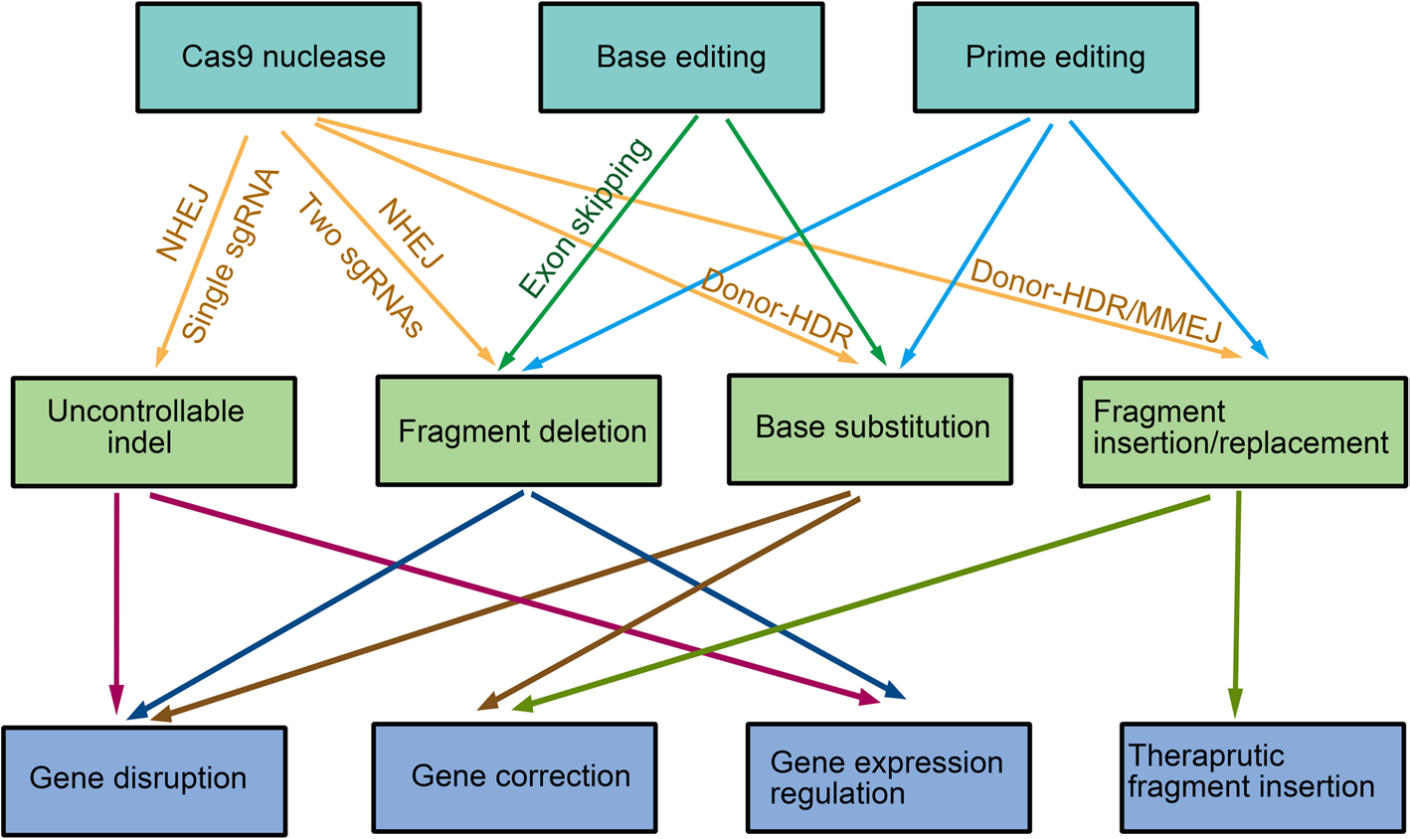
Therapeutic Strategies of Cas9 nuclease, base editor and prime editor. Different Cas9-related editing tools (above) can produce different types of edits to the genome (middle) and thus can cause different changes to the corresponding genes (below)

The versatility of Cas9 nuclease and its derivates has found its applications ranging from basic science to the clinic (Table 2). As discussed above, the versatility of Cas9 system now enabled virtually all types of genome editing, including targeted undesired indels formations, base substitutions, designed fragment deletion or insertions. Such editing strategies can be harnessed to disrupt or restore gene function, regulate gene expression and insert therapeutic DNA fragment, which can be used to treat a diverse set of disorders, including genetic diseases, metabolic diseases, cancer and infectious diseases etc. (Fig. 4).

**Table 2:** Clinical CRISPR/Cas9 therapy in malignant, metabolic and inherited diseases

Editor	Disease	Target gene	Editing outcome	Interventions	Therapeutic principle	References/identifier	Phase/status
CRISPR/Cas9	Lymphoma	PD-1 / TCR	Targeted gene disruption and insertion	Engineered autologous anti-mesothelin CAR-T	Gene disruption and insertion	NCT03545815	Phase 1
						NCT04637763	Phase 1
		TRAC, β2M, and CD70	Targeted gene disruption and insertion	Engineered allogeneic anti-cd70 CAR-T	Gene disruption and insertion	NCT04502446	Phase 1
		HPK1	Targeted gene disruption and insertion	Engineered autologous anti-CD19 CAR-T	Gene disruption and insertion	NCT04037566	Phase 1
	Leukemia and lymphoma	B2M, CIITA and TRAC	Gene disruption and insertion	Engineered Allogeneic Anti-CD19 CAR-T	Gene disruption and insertion	NCT05037669	Phase 1
	Cancer	CISH	Targeted gene disruption	Engineered Tumor-Infiltrating Lymphocytes	Gene disruption	NCT04426669	Phase 1/Phase 2
	Myeloma	TRAC, TRBC and PD-1	Targeted gene disruption and insertion	Engineered autologous anti-NY-ESO-1 TCR-T	Gene disruption and insertion	NCT03399448	Phase 1
	Multiple Myeloma	TRAC and B2M	Targeted gene disruption and insertion	Engineered Allogeneic Anti-BCMA CAR-T	Gene disruption and insertion	NCT04925206	Phase 1
						NCT03166878	Phase 1/Phase 2
				Engineered Allogeneic Anti-cd19 CAR-T	Gene disruption and insertion	NCT04244656	Phase 1
	Renal Cell Carcinoma	TRAC and B2M	Targeted gene disruption and insertion	Engineered Allogeneic Anti-cd70 CAR-T	Gene disruption and insertion	NCT04438083	Phase 1
	B Acute Lymphoblastic Leukemia	CD52 and TRAC	Targeted gene disruption and insertion	Engineered Allogeneic Anti-cd19 CAR-T	Gene disruption and insertion	NCT04557436	Phase 1
	Solid Tumor	PD-1	Targeted gene disruption	Infusions of CRISPR–Cas9 treated cytotoxic T lymphocytes (ex vivo)	Gene disruption	NCT03747965	Phase 1
	Gastric carcinoma, nasopharyngeal carcinoma, T cell lymphoma, adult Hodgkin lymphoma, diffuse large B cell lymphoma	PD-1	Targeted gene disruption	Infusions of CRISPR–Cas9 treated cytotoxic T lymphocytes (ex vivo)	Gene disruption	NCT03044743	Phase 1/Phase 2
	Advanced Hepatocellular Carcinoma	PD-1	Targeted gene disruption	Infusions of CRISPR–Cas9 treated cytotoxic T lymphocytes (ex vivo)	Gene disruption	NCT04417764	Phase 1
	Metastatic non-small cell lung cancer	PD-1	Targeted gene disruption	Infusions of CRISPR–Cas9-treated T cells (ex vivo)	Gene disruption	NCT02793856	Phase 1
	Esophageal cancer	PD-1	Targeted gene disruption	Infusions of CRISPR–Cas9-treated T cells (ex vivo)	Gene disruption	NCT03081715	Not Applicable
	B Cell Leukemia	CD52 and TRAC	Targeted gene disruption and insertion	Engineered Allogeneic anti-CD19 and CD20 or CD22 CAR-T	Gene disruption and insertion	NCT03398967	Phase 1/Phase 2
	T cell malignancies	CD7	Targeted gene disruption and insertion	Engineered Allogeneic anti-CD7 CAR-T	Gene disruption and insertion	NCT03690011	Phase 1
	B-cell Malignancy	TRAC and B2M	Targeted gene disruption and insertion	Infusions of CRISPR–Cas9 treated CD19-directed T cells (CTX110) (ex vivo)	Gene disruption and insertion	NCT04035434	Phase 1
	Sickle Cell Disease	β-globin		autologous hematopoietic stem progenitor cells		NCT04774536	Phase 1/Phase 2
	β-Thalassemia	*BCL11A enhancer*	Targeted enhancer disruption	Engineered Autologous CD34 + Hematopoietic Stem and Progenitor Cells	Regualtion of gene expression	NCT03655678	Phase 2/Phase 3
						NCT04925206	Phase 1
						NCT04208529	Not Applicable
						NCT03745287	Phase 2/Phase 3
						NCT05477563	Phase 3
		*HBB*	Mutation correction	Engineered Autologous Hematopoietic Stem and Progenitor Cells	Restore gene function	NCT03728322	Early Phase 1
	Virus infection (HSV-1)	HSV-1	DNA disruption	corneal injection of CRISPR/Cas9 mRNA targeting HSV-1	Virus genome disruption	NCT04560790	Not Applicable
	Virus infection (COVID-19)	PDCD1 and ACE2	Targeted gene disruption	Engineered T lymphocytes	Gene disruption	NCT04990557	Phase 1/Phase 2
	HIV-1-infection	CCR5	Targeted gene disruption	Engineered Allogeneic CD34 + Hematopoietic Stem and Progenitor Cells	Gene disruption	NCT03164135	Not Applicable
	HPV-related Cervical Intraepithelial Neoplasia I	E6/E7	Targeted gene disruption	Gel with Talen or Cas9 plasmids	Virus genome disruption	NCT03057912	Phase 1
	Leber congenital amaurosis type 10 (LCA 10)	CEP290	Exon skipping	Subretinal injection of AGN-151587 (EDIT-101) (in vivo)	Gene correction	NCT03872479	Phase 1/Phase 2
Base editor	heterozygous familial hypercholesterolemia (HeFH) / atherosclerotic cardiovascular disease (ASCVD) / uncontrolled hypercholesterolemia	pcsk9	Targeted gene disruption	LNP-Base editor mRNA	Gene disruption	NCT05398029	Phase 1
	β-thalassemia (TDT) /sickle cell disease (SCD)	BCL11a	Enhancer disruption	Base edited CD34 + hematopoietic stem	Regulation of gene expression	NCT05456880	Phase 1/Phase 2

### Cas9 nucleases mediated gene editing

#### Cas9 nucleases mediated gene disruption

Since DSBs induced by Cas9s or other nucleases are mainly repaired by the mammalian cells to undesired indels, it is straightforward to harness the nuclease activity of Cas9s for the purpose of gene disruption (Table 3). Targeting gene coding regions, especially those containing conserve functional protein domains, to introduce frame-shifting indels or mis-sense mutation is a frequently used strategy in designing Cas9 based therapy. For example, 1. vascular endothelial growth factor receptor 2 (VEGFR2) is an important therapeutic target for angiogenesis-related disorders such as proliferative diabetic retinopathy and neovascular age-related macular degeneration [220]. Disruption of this receptor by AAV mediated retina expression of Cas9 system efficient abrogates angiogenesis in the mouse models of oxygen-induced retinopathy and laser-induced choroid neovascularization [221]. Another example came from transthyretin amyloidosis, a life-threatening disease stemmed from progressive accumulation of misfolded transthyretin (TTR) protein that mainly produced by the liver. LNP mediated expression of Cas9 system against liver TTR gene efficiently reduced the concentration of TTR protein in serum in both animal model and human patient [222]. In addition, targeting liver pcsk9 by AAV or LLN mediated expression of Cas9 systems significantly reduced plasma cholesterol levels in mice, preventing the genesis or the development of cardiovascular disease [39, 223].

**Table 3:** Representative preclinical CRISPR/Cas9 therapy in inherited disease models

Editor	Disease	Target gene	Editing outcome	Delivery	Therapeutic principle	References
CRISPR/Cas9-knockout	Sickle Cell Disease/β-thalassemia	BCL11a	Enhancer disruption	Electroporation	Gene disruption	[224]
	Duchenne musculardystrophy (DMD)	DMD	Exon skipping	AAV	Restore gene function	[225]
				LNP	Restore gene function	[226]
				NanoMEDIC	Restore gene function	[227]
	Primary hyperoxaluria type I (PH1)	AGXT gene	Targeted gene disruption	AAV	Gene disruption	[228]
		LDH	Targeted gene disruption	AAV	Gene disruption	[229]
	Hearing loss	Htra2	Targeted gene disruption	AAV	Gene disruption	[230]
	Hutchinson-Gilford (HGPS)	LMNA(laminA)	Targeted gene disruption(part)	AAV	Gene disruption	[231]
	Myotrophic lateral sclerosis (ALS)	SOD1(mutant)	Targeted gene disruption	AAV	Gene disruption	[232]
	Cone-rod dystrophy (CORD6)	GUCY2D	Targeted gene disruption	AAV	Gene disruption	[233]
	Cancer	FO	CRISPR/Cas9-knockout	Adv	Gene disruption	[234]
		TGFBR2	CRISPR/Cas9-knockout	Electroporation	Gene disruption	[235]
	HIV-1/AIDS	HIV-1	Targeted gene disruption	AAV	Gene disruption	[236]
CRISPR/Cas9-HDR	Sickle Cell Disease/β-thalassemia	HBB	Mutation correction	AAV	Restore gene function	[237]
	Phenylketonuria (PKU)	PAH	Mutation correction	AAV	Restore gene function	[238]
	Atherosclerosis	LDLR	Mutation correction	AAV	Restore gene function	[239]
	Hemophilia B	FIX	gene knock-in	AAV	Restore gene function	[240]
	OTC deficiency	OTC	Mutation correction	AAV	Restore gene function	[44]
	Cancer	AAVS1	gene knock-in	Electroporation	Restore gene function	[241]
		TRAC	gene knock-in	AAV	Restore gene function	[241]
Base editor	Sickle Cell Disease/β-thalassemia	HBG	promotor mutation	HDadv	Restore gene function	[242]
	Phenylketonuria (PKU)	PAH	Mutation correction	AAV	Restore gene function	[243, 244]
				LNP	Restore gene function	[245]
	Duchenne musculardystrophy (DMD)	DMD	Mutation correction	AAV	Restore gene function	[190]
	Hearing loss	TMC1	Mutation correction	AAV	Restore gene function	[246]
	Hutchinson-Gilford (HGPS)	LMNA	Mutation correction	AAV	Restore gene function	[247]
	Atherosclerosis	pcsk9	Targeted gene disruption	LNP	Gene disruption	[248]
			Targeted gene disruption	eVLP	Gene disruption	[249]
	Myotrophic lateral sclerosis (ALS)	SOD1(mutant)	Targeted gene disruption	AAV	Gene disruption	[232]
	Leber congenital amaurosis (LCA)	RPE	Mutation correction	AAV	Restore gene function	[250]
				eVLP	Restore gene function	[249]
	Cancer	CD52, CD7, PD1, and TCRα(CAR T cell)	Targeted gene disruption	Electroporation	Gene disruption	[251]

Indel formation can also be harnessed to disrupt the regulator elements of genes, such as splicing donors or acceptors, transcription enhancers or suppressors and so on. BCL11a is an attractive target since its loss-of-function releases the repression of fetal γ-globin, which in turn can rescue the phenotype of beta-globin mutation related anemia [252]. Targeting the enhancer element of BCL11a or its binding sites within γ-globin locus are frequently used strategy to reactivate the expression of the latter [224, 252–254]. Delivery of CRISPR/Cas9 system targeting bcl11a enhancer into CD34 + hematopoietic stem cells via in vitro electroporation is an effective way to reactivate the expression of γ-globin in red blood cells [50, 224]. Recently, such strategy has achieved positive outcomes in human patients [50, 224]. The infusion of edited HSC increased the level of fetal globin, reduced the requirement of blood transfusion in in β-thalassemia and the incidence of vaso-occlusive episodes in sickle cell disease [50, 224].

Besides producing undesired indels, Cas9 nuclease activity can also be rewired to produce targeted fragment deletions by coupling with dual sgRNAs against same loci [225]. This feature can be used to delete genomic fragment that contains mutations locating in the redundant sequences but impaired the expression of that gene. For example, DMD gene that encodes a muscle nutrition protein, dystrophin, contains 24 functional redundant spectrin-like repeats, within which mutations may impair RNA splicing or translation (pre-mature codon) thereby disrupting the expression of DMD and leading to muscle mass loss [255]. Dual sgRNA strategy has been demonstrated to be efficient in deleting the mutant exon and restore DMD gene expression and function [225]. Targeted fragment deletion can also be used to delete mis-spliced exons that contained stop codons and were resulted from mutations to splicing elements, such as cis -regulatory elements, core spliceosomal components and trans -acting regulatory factors**.** For example, Lu et al. used dual sgRNA strategy to deleted mis-spliced exons that contained stop codon in humanized ß-globin IVS-2 mouse model, resulting in the correction of abnormal ß-globin RNA splicing and improved β-thalassemia related syndrome [256]. A type of Leber congenital amaurosis 10 (LCA10), a rare inherited retinal dystrophy, is also caused by mis-spliced exon, which is stemmed from CEP290 IVS26 mutation [257]. Ruan et al.used AAV to deliver Cas9 and dual sgRNAs into the retinas of CEP290 IVS26 mutant mice and observed efficient targeted deletion of the mutant fragment. The deletion restored the expression of CEP290 protein and significantly rescued LCA related phenotypes [258].

#### Cas9 nucleases mediated gene konck-in

As mentioned above, DSBs can also be fixed via HDR pathway when sufficient repair template donors were provided. Therefore, Cas9 nuclease can be used to correct gene mutations or insert therapeutic fragment into the target region (Table 3). For example, Yang et al. developed a dual AAV system enabling the HDR-mediated in vivo correction of point mutation of OTC gene in mouse hepatocytes [44]. In this report, they infused two AAVs into neonatal or adult mice, one expressing Cas9 and the other expressing a guide RNA and the donor DNA. The infusion resulted in correction of the mutant OTC gene in about 10% hepatocytes and increased survival rate in neonatal mice but not in adult mice, which is possibly due to that HDR activity is limited to S/G2 phases of proliferating cells (Regulation of homologous recombination in eukaryotes. Enrichment of G2/M cell cycle phase in human pluripotent stem cells enhances HDR-mediated gene repair with customizable endonucleases.) [44]. Richards et al. also used AAV to deliver Cas9-mediated HDR editing system in to PKU mouse model and corrected the Pah point mutation in liver cells, partially restoring PAH activity and significantly reducing the level of blood phenylalanine [238]. Zhao et al. used similar strategy to correct a non-sense mutation of Ldlr gene in mouse model and observed partial rescue of LDLR protein expression and function, which significantly reduced the serum levels of total cholesterol, total triglycerides and LDL-cholesterol [239]. Besides correcting point mutation, Cas9-mediated HDR strategy can also be used to targeted insert a therapeutic DNA fragment into desired locus. In most cases, the inserted fragments were designed to therapeutic genes. For example, Lisjak et al. used this strategy to insert the coding region of human coagulation factor IX (HFIX) into albumin locus in hepatocytes in vivo, enabling the co-expression of HFIX with albumin. The ectopically expressed HFIX successfully rescued hemophilia related phenotypes [240]. Eyquem et al. used similar strategy to insert CD19-specific CAR into the T cell receptor α constant (TRAC) locus of T lymphocytes to allow the expression of CD-19 CAR under the control of TRAC promoter [241]. Besides HDR, another DNA repair mechanism, MMEJ, can also be used to insert therapeutic fragment. Compared with HDR that usually requires homologous arms of more than 500 bp, MMEJ strategy only requires 20–30-bp homologous arms, which significant simplifies the construction of donors and reduces size of donor DNA [205]. By using MMEJ strategy, Yao et al. inserted a full length fumalacetacetic acid hydrolase 1 (HT1) gene into the hepatocytes of HT1 deficient mice to rescue the liver damage phenotype [259] (Table 3).

### Therapeutic applications of base editors

However, because NHEJ activity is prominent in mammalian cells, HDR or MMEJ strategy produced more unwanted indels than desired edits in most cases, making it not so efficient and safe for clinical applications. By contrast, base editors are more precise and efficient in introducing single base conversions. Such a feature is particularly useful for correcting point mutations or create desired functional base conversions (Table 3). For example, introducing missense or non-sense mutations within gene coding region can lead to either gain- or lose-of-function of target genes. Chadwick et al. delivered cytosine base editor to the liver of adult mice to generate stop codon within pcsk9 gene and observed significant reduction in plasma PCSK9 protein and cholesterol levels, demonstrating the therapeutic potential of base editing in silencing gene function [260]. More straightforwardly, base editors can also be used to correct diseases related point mutations. Rossidis et al. delivered cytosine base editor into the uterus of pregnant tyrosinemia type 1 mutant mice to correct Fah mutation [261]. They showed that in-utero delivery of base editor successfully rescued the lethal phenotype of neonatal Fah mutant mice, demonstrating the potential of in utero base editing in the treatment of genetic diseases causing in prenatal or neonatal death [261]. Zhou et al. and Villiger et al. delivered cytosine base editors derived from SaCas9 into the liver of neonatal and adult PKU mice respectively [243, 244]. They showed that AAV-8 delivered SaCas9 base editor efficiently corrected PAH point mutation and reduced the plasma phenylalanine to physiological level [243, 244]. In addition, base editing can also be used to manipulate RNA splicing via converting key bases within splicing regulatory elements. Li et al. used a non-popular cytosine base editor developed by themselves, eTAM, to destroy DMD splicing site, leading the skip of 4^th^ exon that contains frame-shifting mutation, which genetically restored the open reading frame of DMD gene [262]. They showed that a single-dose of AAV9-eTAM achieved > 50% targeted exon skipping in the Dmd mRNAs and restored up to 90% dystrophin protein in the heart, leading to an increased life span of the Dmd mutant mice [262].

## Delivery of Cas9 and its derivates

CRISPR-Cas9-mediated gene editing tools are very powerful, revolutionizing the field of gene therapy and showing encouraging results in a variety of applications. However, the in vivo delivery of editors remains a critical limitation for these editing tools to treat specific diseases. In vivo delivery methods of gene editing tools are classified into two main categories: viral and non-viral systems.

### Viral delivery systems

Viruses have evolved naturally to deliver nucleic acids in vivo to different cell types, which makes them interest targets for delivering exogenous genes. Currently, several types of viral vectors have been developed and modified for the delivery of gene editing tools [263]. Among them, adeno-associated virus (AAV), as well as adenovirus have been widely used in preclinical studies of Cas9-mediated gene editing strategies [263] (Table 3).

#### AAV delivery

AAV is a single-stranded DNA virus, the smallest and simplest animal virus in the family Microviridae. AAV particles are envelope-free and consist of an icosahedral protein capsid of approximately 25 nm in diameter and a single-stranded DNA genome of ~ 4.7 kb [264]. Compared with other viral delivery systems, AAV has the advantages of low immunogenicity, high efficiency and high biocompatibility [265]. And importantly, unlike lentivirus and retrovirus, recombinant AAVs very rarely integrate into host genomes [266–268]. In addition, there are plenty of different types of capsid serotypes, directing AAVs to different target tissues in vivo [269]. These features nominate AAVs as promising gene therapy vehicles [270].

However, the small packaging capacity of AAVs is a major limitation for their application. AAVs have a packaging capacity of about 5 kb, including the transgene cascade and two flanking inverted terminal repeats (ITR) [271, 272]. Therefore, the room for exogenous transgene expression cascade is limited to ~ 4.7 kb, which limits them as delivery tools for Cas9-related gene editing, since Cas9 proteins are generally large. The most widely used Cas9, SpCas9, is about 4.3 kb, and its derivates, such as BE and PE, are even larger [22, 27, 30].

To overcome size limitations, researchers have developed several methods that allow Cas9-mediated gene editing tools to be packaged into AAV vectors for in vivo delivery. Several groups have developed strategies to split Cas9 and its derivates into two halves. Intein mediated protein splicing is the most frequently used splitting system. By optimizing the splitting sites, the efficiencies of split Cas9 systems are similar to or only slightly lower than those of their full-length equivalents, enabling practicable AAV mediated in vivo genome editing. For example, David Liu's group have designed a double AAV base editing strategy to treat the Hutchinson-Gilford premature aging syndrome (HGPS) mouse model and corrected the C·G-to-T·A mutation in the LMNA gene, achieving up to 30% gene correction efficiency in heart tissue [247]. Schwank and colleagues used a dual AAV9 strategy to deliver ABE targeting Pcsk9 to mice, achieving 60% base editing in the liver and significant reductions in serum pcsk9 protein levels as well as serum cholesterol levels [248]. Such strategy has also been used in central nervus tissue to knock out mutated Huntington (HTT) genes to correct pathogenic mutations in a mouse model of Niemann-Pick disease [273].

Alternatively, using smaller Cas9 can achieve single AAV delivery. For example, Cas9 from Staphylococcus aureus (SaCas9) is commonly used for single AAV transduction because its size is only 3.2 kb [112]. Such a compact size endows it to be co-packaged with the sgRNA expression cascade in a single AAV. Zhang and colleagues used a single AAV system to in vivo deliver SaCas9 to efficiently knock out Pcsk9 and lower serum cholesterol in mice [39]. In addition, an ongoing clinical trial uses a single AAV for subretinal delivery of SaCas9 double sgRNA has been lunched to delete a pathogenic mutation in the CEP290 gene in patients with Leber congenital amaurosis 10 [258]. In addition to SaCas9, many other compact Cas9 variants were identified and characterized, such as Nme2Cas9 (3.24 kb, PAM = N4CC) [96, 102, 274–276], CjCas9 (2.95 kb, PAM = N4RYAC) [70, 277, 278] and SauriCas9 (3.18 kb, PAM = N2GG) [82], all of which are capable of single AAV delivery. These Cas9 variants also broaden PAM recognition and expand the targeting range of the single AAV gene editor.

#### Adenoviral delivery

Adenovirus (Ad) is an icosahedral envelope-free virus, 90–100 nm in size, with a large (36 kb) genome [279]. Ad is the most commonly used viral vector (> 20%) in gene therapy clinical trials, possibly due to its large packaging capacity, genetic stability, high transduction efficiency, and ease of production [279]. Currently, there are 57 known Ad serotypes that can infect human and 100 serotypes that can infect primates, allowing researchers to modulate Ad tissue targeting by using different capsids [249]. In 2017, Musunuru and colleagues used Ad to deliver CBE into mice and observed a 28% editing efficiency of Pcsk9, successfully lowering plasma cholesterol levels in treated mice [260]. Lieber and colleagues used Ads to introduce ABE into HSC in vivo to disrupt the blocker binding site in the fetal hemoglobin promoter, significantly upregulating fetal hemoglobin [280]. Ad has also recently been used for in vivo introduction of Prime editor. Schwank and colleagues used Ad to deliver PE2 without the RNaseH structural domain into neonatal or adult mice and observed 58% and 36% editing efficiency, respectively [281]. Although adenoviruses can introduce gene editing tools in vivo and produce effective editing, their dosages are limited during application, possibly due to their high immunogenicity and cytotoxicity.

### Non-viral delivery

In addition to the modification of existing viral vectors, other types of delivery systems can be used for transient in vivo delivery of exogenous genes, such as non-viral vectors (Table 3). Among them, lipid nanoparticles (LNP) are the most frequently used non-viral gene delivery systems, holding great potential for in vivo applications of gene editing tools. Besides, virus-like particles (VLP) are also developed to deliver these tools.

#### LNP delivery

Lipid nanoparticles (LNPs) have been used for decades to deliver nucleic acids, including siRNA and therapeutic mRNA, and in recent years LNPs have been widely used for in vivo delivery of gene editing tools [222, 226]. In most cases, these tools were delivered in the form of RNA, which is a negatively charged hydrophilic macromolecule and is electrostatically repelled by the same electrical properties as the cell membrane, making it difficult to enter the cell and susceptible to rapid degradation by the ubiquitous ribonuclease (RNase) [282]. Therefore, RNA needs a protective layer to "transport" it into the cell. Since cell membranes are mainly composed of lipids, liposome coating can make it easier for RNA to pass through the membrane and be released into the cytoplasm. To achieve this, liposomes require a positively charged lipid molecule that can attach to negatively charged RNA, in addition to structural lipids (which mimic the cell membrane and shield the positive charge) and poly (ethylene glycol)-anchored lipids (which prevent aggregation of LNPs and side reactions with the biological environment). Therefore, LNPs are typically composed of four molecules: cationic or ionizable lipids, co-lipids, poly (ethylene glycol) -lipids, and cholesterol [283].

Compared with cationic lipids, ionizable lipids (ILs) are less toxic and more effective in in vivo delivery [284]. When formulated as LNPs, ILs are designed to display electroneutrality at physiological pH, but behave positively charged in vivo in acidic LNP nuclei. This pH-adapted ionization results in enhanced efficacy with reduced toxicity, making them more suitable for nucleic acid delivery [284]. These lipids typically make up 30–50% of the total lipids in the formulation [285]. Cholesterol, a naturally abundant component of cell membranes, is one of the so-called structural lipids found mainly in the outer shell of LNPs and typically accounts for 20–50% of the total lipids in LNPs [285]. Phospholipids contribute to the encapsulation of nucleic acids and the stability of LNPs and usually represent only 10—20% of the total lipids in the formulation [285]. Phospholipids are used as structural lipids because they can spontaneously organize into lipid bilayers and have a high phase transition temperature thus ensuring the membrane stability of LNPs. Phospholipids are located at the periphery of LNPs, just like cell membranes, and PEG lipids are an important component in controlling the half-life and cellular uptake of LNPs [286]. During LNP assembly, PEG chains are located in the outer shell of the nanoparticles due to their hydrophilicity and large volume. PEG provides an external polymer layer for LNPs to hinder the adsorption of serum proteins and mononuclear phagocyte systems and prolong the in vivo circulation. PEG also prevents aggregation of nanoparticles during storage as well as in the blood [286]. Changing the properties of these components can produce LNPs with different properties, including different pharmacokinetic profiles and the ability to target different cell types.

The key advancement of the LNP formulation is that it enables efficient packaging and delivery of mRNA, allowing LNP to be widely used for in vivo delivery of exogenous mRNA, including in vivo introduction of Cas9-mediated gene editors. Anderson and colleagues co-packaged SpCas9 mRNA and chemically modified sgRNA via LNP and injected intravenously into mice, targeting Pcsk9 produced 80% editing efficiency and reduced and serum Pcsk9 to undetectable levels [287]. This result demonstrates for the first time the promise of therapeutic level gene editing in mice by LNP encapsulating both Cas9 nuclease mRNA and chemically modified sgRNA. Since then, researchers have shown that LNP can successfully introduce Cas9 in complex with sgRNA into crab-eating monkeys, which can achieve 73% TTR destruction in the liver and a corresponding reduction of serum TTR protein by more than 94% [288]. Next, clinical data showed that LNP introduced Cas9-sgRNA complex into patients could also reduce their serum TTR levels by up to 87% with low off-target editing. These results confirm the possibility of in vivo genome editing by LNP introduction of Cas9 and sgRNA complexes. In addition to delivering Cas9 nuclease mRNA, LNP has also been used to deliver base editor mRNA into the liver of mice and non-human primates. Xue and colleagues observed that delivery of ABE mRNA by LNP into the liver of a mouse model of tyrosinemia yielded a 12.5% base editing efficiency [289]. Schwank and colleagues observed a 10% base editing rate of phenylalanine hydroxylase mutations in the liver of mice with phenylketonuria model by delivery of LNP encapsulating SaCas9-BE3 mRNA [243]. Recently, it was demonstrated by Kathiresan, Schwank et al. that the splice site of PCSK9 can be effectively interrupted by LNP delivered ABE mRNA into the liver of mice as well as crab monkeys, thus disrupting the expression of functional PCSK9 protein. Accompanied with the delivery, substantial (90%) and sustained (> 8 months) inhibition of serum PCSK9 protein and 60% reduction in blood cholesterol were achieved in crab monkeys [248]. The above studies suggest that the LNP delivered base editor has high clinical therapeutic potential for liver related genetic or metabolic diseases.

Since most LNPs injected intravenously naturally accumulate in the liver, editing of other organs outside the liver is challenging. Dahlman and colleagues developed a strategy to screen hundreds of different LNPs simultaneously in vivo [290]. The strategy uses unique DNA barcodes to label different LNP formulations, allowing the LNPs to be traced by sequencing the barcodes [290]. Using this strategy, Dahlman and colleagues found an LNP that could target spleen endothelial cells. They also showed that this LNP could efficiently deliver Cas9 mRNA and sgRNA to mouse spleen endothelial cells, yielding editing efficiencies comparable to those of hepatocytes [291]. In addition, Siegwart et al. developed selective organ-targeted (SORT) LNPs by supplementing an additional charged lipid component to modulate the internal charge while without significantly disrupting the standard four-component nature of LNP [292, 293]. They found that altering the charge and concentration of this additional component was sufficient to redirect LNP to the lung or spleen. These SORT LNPs successfully delivered Cas9 mRNA and sgRNA specifically to the lungs of mice, achieving 15% lung tissue editing [292]. These studies have shown that the targeting of LNPs can be modulated by changing their composition.

In addition, besides systematic delivery, local injection has been demonstrated to be effective in several organs. A panel of pre-clinical studies have shown that local injection of lipid-coated RNP into the inner ear and retina of mice can be effective for nuclease editing and base editing [246].

### Virus-like particle (VLP) delivery

In addition to LNP, the Virus-like Particle (VLP) are also capable of delivering gene editing tools. These particles are non-infectious viral protein assemblies that package the desired mRNA, protein or RNP for delivery to the appropriate tissues in vivo [294]. Because VLPs are derived from existing viral backbones or viral capsid like proteins, they have the similar delivery properties to their corresponding viruses, including cargo encapsulate, endosome escapement, and the ability to be reprogrammed to target different cell types [294]. However, unlike viruses, VLPs transiently deliver gene editors in the form of mRNA or RNP, which reduces the risk of off-target gene editing and viral genome integration [295].

Almost all reported VLP architectures for delivering mRNA or protein cargo are based on retroviruses because retroviruses have several characteristics that are well suited for VLP [296]. Immature retroviral particles are spherical and typically lack rigid structural symmetry, which allows higher loading flexibility as compared with non-enveloped icosahedral viruses. In addition, the large particle size of retroviruses (100–200 nm) provides more physical space for packaging large proteins such as Cas9 [296]. Finally, retroviruses are inherently modular in terms of cellular targeting and packaging [297]. Cell type specificity is determined by the envelope glycoprotein, while packaging is controlled by the capsid protein [297]. This modularity suggests that the VLP capsid structure, which effectively packages the desired "cargo", can easily bind to a variety of existing envelope glycoproteins to modulate targeting specificity.

David Liu's group has recently developed an engineered VLP (eVLP) based on Moloney MLV (MMLV) that greatly optimizes the protein packaging and delivery capabilities of the VLP [249]. eVLP can efficiently package Cas9 nuclease or base editors RNP and mediate effective therapeutic levels of gene editing in multiple organs of mice. Local injection of eVLP into mouse brain successfully delivered base editor RNP in vivo, resulting in robust base editing in VLP-rich transduced cells (60% editing) [249]. Subretinal injection of eVLP into a mouse model of genetic blindness effectively corrected the causative point mutation and improved visual function. Finally, a single intravenous injection of eVLP into mice achieved 63% editing of Pcsk9 in the liver and 78% knockdown of serum Pcsk9 levels, which is comparable to the efficiency that produced by AAV and LNP delivery [249]. VLP from lentivirus has also been developed to package Cas9 mRNA and sgRNA, which has been demonstrated to produce therapeutic level of genome editing when locally injected [298, 299]. VLP engineered from mammalian retrovirus-like protein PEG10 are also capable of delivering Cas9 mRNAs and sgRNAs, producing robust editing in vitro (~ 60%) [300]. Together, these investigations demonstrated the potential of VLPs for therapeutic delivery of gene editing tools.

## Perspectives and challenges

The past ten years have witnessed the explosion of genome editing technology, with more and more Cas9 derived genome editing tools discovered and evolved. The ability of those tools to efficiently and precisely install a virtually wide range of changes to the genome has revolutionized the research of both basic life science and clinical medicine. In particular, these tools have provided fantastic new direction for the field of gene and cell therapy.

Noteworthy, many challenges need to be addressed before genome editing could be widely used in human patients. One of the major challenges would be safety issue. Nearly all editing tools have been reported to produce more or less levels of off-target or undesired editing, ranging from base substitutions or small scale indels (several to tens of base pairs) to large scale fragment deletions (hundreds to millions of base pairs) and even chromosomal scale variations in either sequence dependent or independent manner. Such unintended deleterious outcomes should be systematically evaluated when designing therapeutic strategies. Fortunately, several strategies have been developed to reduce such off-target effects, including high-specific and truncated sgRNAs, high-fidelity Cas9 variants, base editors with internally inlaid architecture or with engineered deaminases harboring hypoactive mutations. Alternatively, modulating endogenous pathways have also been4shown to reduce several types of undesired editing outcomes. Enhancing histone acetylation by HDAC inhibitors has been shown to improve product purity of cytosine base editors by strengthening the interaction between UGI and UNG. Inhibition of DNA mismatch repair pathway has been shown to reduce the frequency of prime editor produced unwanted indels. Another challenge came from the pre-existing adaptive immune responses to the Cas9 proteins. Since currently the most powerful and commonly used Cas9 proteins, SpCas9 and SaCas9, are derived from bacterial species that are prevalent in human, about 50% population harbor pre-existing immune response to them. Study from monkey revealed that such pre-existing immune response would eliminate the genome-edited cells. In addition, it was found that Cas9 delivered by AAV produces higher immune response than that delivered by LNP, suggesting that a transient expression of Cas9 is optimal to avoid the already existing Cas9 immune response. Moreover, since repeated administration of Cas9 in monkeys produces a stronger immune response than the initial administration, it is highly recommended to achieve the therapeutic goal with a single dose. Additionally, in vivo delivery of genome editors to target cells is another big challenge, especially considering that Cas9 derived editors are large in size. Both viral and non-viral delivery systems have been used for in vivo delivery of genome editors. However, viral systems, such as AAV, usually express the editors for a long term. Under such condition, sustained activity of the editor is supposed to amplify genotoxic effects. Moreover, long-term expression also increases the chances for the target cells to be cleared by immune system. Non-viral systems, especially lipid nano-particles, are efficient in delivering transgenes to several organs including liver, lung and spleen. Recently, LNP delivered Cas9 based editors has achieved significant success in manipulating liver related targets, such pcsk9, TTR and PAH. But as for organs other than the liver, LNP were not so efficient. Tremendous efforts have been made aiming to overcome these challenges and fill the gap between basic research and clinical translation by developing new editing tools, editing strategies and delivery systems. Hopefully, these efforts will speed up the therapeutic application of genome editing in the near future.  

## Data Availability

Not applicable.
